# Functional diversity of three tandem C-terminal carbohydrate-binding modules of a β-mannanase

**DOI:** 10.1016/j.jbc.2021.100638

**Published:** 2021-04-07

**Authors:** Marie Sofie Møller, Souad El Bouaballati, Bernard Henrissat, Birte Svensson

**Affiliations:** 1 Department of Biotechnology and Biomedicine, Technical University of Denmark, Lyngby, Denmark; 2 Architecture et Fonction des Macromolécules Biologiques, CNRS, Aix-Marseille Université, Marseille, France; 3 Department of Biological Sciences, King Abdulaziz University, Jeddah, Saudi Arabia

**Keywords:** protein–carbohydrate interaction, multimodular enzyme truncation, substrate specificity, enzyme kinetics, galactomannan, glucomannan, mannan, affinity gel electrophoresis, pull-down assays, GFP–domain fusion, AGE, affinity gel electrophoresis, BMCC, bacterial microcrystalline cellulose, CBM, carbohydrate-binding module, CBM10, CBM family 10, CD, catalytic domain, CGM-lv, low-viscosity carob galactomannan, DNS, 3,5-dinitrosalicylic acid, GG, guar gum, GH, glycoside hydrolase, GH5_8, GH family 5 subfamily 8, HEC, hydroxyethyl cellulose, INM, ivory nut mannan, KGM, konjac glucomannan, LPMO, lytic polysaccharide monooxygenase, PASC, phosphoric acid–swollen cellulose

## Abstract

Carbohydrate active enzymes, such as those involved in plant cell wall and storage polysaccharide biosynthesis and deconstruction, often contain repeating noncatalytic carbohydrate-binding modules (CBMs) to compensate for low-affinity binding typical of protein–carbohydrate interactions. The bacterium *Saccharophagus degradans* produces an endo-β-mannanase of glycoside hydrolase family 5 subfamily 8 with three phylogenetically distinct family 10 CBMs located C-terminally from the catalytic domain (*Sd*GH5_8-CBM10x3). However, the functional roles and cooperativity of these CBM domains in polysaccharide binding are not clear. To learn more, we studied the full-length enzyme, three stepwise CBM family 10 (CBM10) truncations, and GFP fusions of the individual CBM10s and all three domains together by pull-down assays, affinity gel electrophoresis, and activity assays. Only the C-terminal CBM10-3 was found to bind strongly to microcrystalline cellulose (dissociation constant, *K*_*d*_ = 1.48 μM). CBM10-3 and CBM10-2 bound galactomannan with similar affinity (*K*_*d*_ = 0.2–0.4 mg/ml), but CBM10-1 had 20-fold lower affinity for this substrate. CBM10 truncations barely affected specific activity on carob galactomannan and konjac glucomannan. Full-length *Sd*GH5_8-CBM10x3 was twofold more active on the highly galactose-decorated viscous guar gum galactomannan and crystalline ivory nut mannan at high enzyme concentrations, but the specific activity was fourfold to ninefold reduced at low enzyme and substrate concentrations compared with the enzyme lacking CBM10-2 and CBM10-3. Comparison of activity and binding data for the different enzyme forms indicates unproductive and productive polysaccharide binding to occur. We conclude that the C-terminal-most CBM10-3 secures firm binding, with contribution from CBM10-2, which with CBM10-1 also provides spatial flexibility.

Interactions between proteins and carbohydrates play a vital role in life and have through evolution been optimized to match the environments where they take place. Affinities of protein–carbohydrate complexes range from millimolar to nanomolar. Low-affinity binding (millimolar range) is considered a key factor in dynamic systems, such as plant cell wall synthesis and degradation, microbe–host interplay, and synthesis and mobilization of storage polysaccharides. The carbohydrate active enzymes catalyzing these different reactions very often contain one or more noncatalytic carbohydrate-binding modules (CBMs) that facilitate the formation of enzyme–substrate complexes and can specifically bind with the substrate polysaccharides or with different polysaccharides located nearby, such as in the plant cell wall. It is common that polysaccharide hydrolases contain several CBMs, but it is difficult to understand the function of these individual CBMs because of their interplay with each other, substrate, other polysaccharides, and the catalytic domain (CD), and perhaps even more than one CD is present (1). Certain insights are established, for example, on enzymes in cellulosomes with domains of the polyspecific CBM32 family interacting with galactose, lactose, polygalacturonic acid, and *N*-acetyllactosamine (2). There are many examples where a functional role has been depicted for specific domains, while being unknown for others in multidomain architectures (3). Clearly, there are relatively few three-dimensional structures available of the more complex multidomain enzymes because of a number of challenges. One being the recombinant production of multimodular proteins containing interdomain linker regions prone to limited proteolysis, another is the dynamic flexibility inherent to the multimodular domain architectures (2, 3).

Mannan polysaccharides (Fig. 1) are found in cell walls of most plants and as storage polysaccharides in seeds and tubers (4). The four prominent types of β-mannans include galactomannans having a β-1,4 mannose backbone with different extents of α-1,6 galactose decoration, glucomannans with an essentially linear backbone of glucosyl and mannosyl units, which in the galactoglucomannans are substituted by α-1,6-galactose (5, 6); essentially linear β-mannans are found in plant stems and as highly packed storage polysaccharides (7, 8) (Fig. 1). Some mannans are acetylated to varying degree depending on the type and botanical source (9).

**Figure 1:**
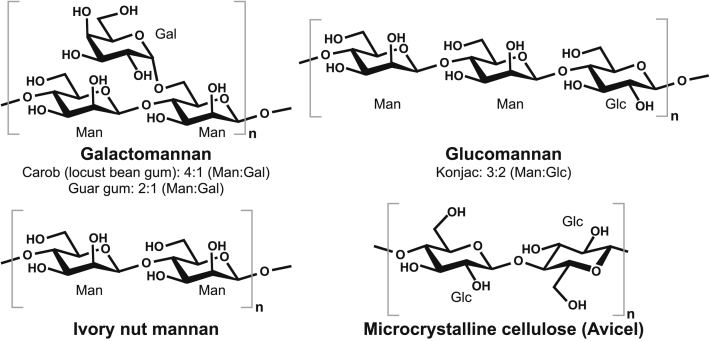
**Structures of the included polysaccharides.** Mannose (Man), galactose (Gal), and glucose (Glc) ratios are given for galactomannans and glucomannan.

Mannans are depolymerized by endo-β-mannanases found in glycoside hydrolase (GH) families GH5, GH26, GH113, and GH134 (10), some of which also contain CBMs facilitating contact with the substrates. Several applications are reported of endo-β-mannanases, for example, for production of oligosaccharides and in saccharification of plant biomass and lignocellulose degradation, where CBMs are of potential importance (11, 12, 13, 14).

CBMs are classified into 88 families based on amino acid sequence similarity (CAZy, database of Carbohydrate Active enZYmes: http://cazy.org) (10). CBM family 10 (CBM10), despite the very small size of 29 to 42 amino acids, presents a diversity of carbohydrate-binding specificities and affinity ranges (Table 1) (15, 16, 17, 18, 19, 20, 21). CBM10s are thus associated with different CDs in plant cell wall polysaccharide-degrading enzymes, including cellulases (22, 23, 24, 25, 26, 27, 28), xylanases (15, 19, 26, 29), mannanases (17, 18, 26, 30, 31, 32, 33, 34, 35), and lytic polysaccharide monooxygenases (LPMOs) (20). Typically, these enzymes have more than one CBM10 or contain also CBMs from other families, CBM2s being particularly common (Table 1).

**Table 1:** Summary of characteristics of enzymes having one or more CBM10(s)

Organism	GenBank accession no.	Modular organization	CBM10 binding	Enzymatic activity	Binding-site residues	Disulphide bridges	Ref.
*C. japonicus*	ACE85176	CBM10-GH26	ND	■◆	DWF	2	(17)
*S. degradans* (*Sd*_GH5_CBM10-1)	ABD79918	GH5_8-CBM10-CBM10-CBM10	■(*K*_*d*_ = 3.7 g/l) [◯•]	■□◆	SWY	2	
*C. japonicus* (*Cj*_AA10_CBM10)	ACE84760	AA10-CBM10	◯(*K*_*d*_ = 7.5–17.3 μM)	◯[•]	YWN	2	(20, 24)
*A. bacterium*	AIF91534	GH5_8-CBM10-CBM10-CBM10	ND	■	YWY	2	(26)
*C. japonicus*	ACE82655	CBM5-CBM10-CBM35-GH5_7	ND	■□◆	YWW	2	(17)
*C. japonicus*	ACE82688	CBM2-CBM10-GH45	◯	•▲	YWY	2	(23)
*C. mixtus*	CAA88761	GH11-CBM60-CE4-CBM10	ND	▲[•]	WWW	2	(29)
*A. bacterium*	AIF91529	CBM10-CBM10-GH10_4	ND	▲	YWW	2	(26)
*C. japonicus*	ACE84673	GH5_8-CBM10-CBM10	◯□[■•]	■◆[□•]	YWW	2	(17)
*C. japonicus*	ACE85978	CBM2-CBM10-GH6	ND	◯[•▲]	YWW	2	(24)
*C. japonicus*	ACE82870	CBM2-CBM10-GH5_53	◯(CBM2+10?)	•[◯▲]	YWY	2	(25)
*C. japonicus* (*Cj*_GH10_CBM10)	ACE85439	CBM2-CBM10-GH10	◯(*K*_*d*_ = 3.9 μM) [•]	•▲	YWW	2	(15, 16, 21, 37)
*C. japonicus*	ACE84076	GH5_2-CBM10-CBM2	◯[▲](CBM2+10)	◯•▲[■]	YWW	2	(22, 24)
*C. japonicus*	AAO31760	GH5_8-CBM10-CBM2	◯□[■•]	■◆[□•]	YWW	2	(17)
*C. japonicus* (*Cj*_GH74_CBM10)	ACE84745	GH74-CBM10-CBM2	◯(*K*_*d*_ = 1.5 μM)[▲]	▲•[■◆]	YWW	2	(19)
*C. japonicus* (*Cj*_GH9_CBM10)	ACE85757	GH9-CBM10-CBM2	◯	ND	YWW	2	(39)
*S. degradans* (*Sd*_GH5_CBM10-3)	ABD79918	GH5_8-CBM10-CBM10-CBM10	■(*K*_*d*_ < 0.13 g/l) ◯(*K*_*d*_ = 1.5 μM)[•]	■□◆	YWW	2	
*A. bacterium*	AIF191551	CBM10-CBM10-CBM60-GH10_4	ND	▲	YWW	2	(26)
*A. bacterium*	AIF91559	GH11-CBM60-CE4-CBM10	ND	▲	YWW	2	(26)
*C. japonicus*	ACE84179	GH11-CBM60-CE4-CBM10	◯[▲]	▲[•]	WWW	2	(29)
*A. bacterium*	AIF91534	GH5_8-CBM10-CBM10-CBM10	ND	■	YFW	2	(26)
*A. bacterium*	AIF91551	CBM10-CBM10-CBM60-GH10_4	ND	▲	YWY	2	(26)
*A. bacterium*	AIF91529	CBM10-CBM10-GH10_4	ND	▲	YWY	2	(26)
*A. bacterium*	AIF91557	CBM2-CBM10-GH5_53	ND	◯•	YWW	2	(26)
*A. bacterium*	AIF91536	GH5_2-CBM10-CBM2	ND	•	YWW	2	(26)
*A. bacterium*	AIF91534	GH5_8-CBM10-CBM10-CBM10	ND	■	YWY	2	(26)
*S. degradans*	ABD81F896	CBM2-CBM10-GH5_4	◯(Low)	◯•	YWW	2	(21, 27)
*T. turnerae*	ABS72374	GH5_2-CBM5-CBM10-GH6_4	◯(Low), chitin	•▲[◯]	YWW	2	(21, 28)
*S. degradans* (*Sd*_GH5_CBM10-2)	ABD79918	GH5_8-CBM10-CBM10-CBM10	■(*K*_*d*_ = 0.4 g/l) [◯•]	■□◆	TWW	1	
*B. animalis* (*Ba*_GH5_CBM10)	ACS46797	GH5_8-CBM10	■(*K*_*d*_ = 0.31 g/l)[□◯•]	■□◆[◯]	TWW	1	(18)
*Vibrio*	BAA25188	GH5_8-CBM10-CBM10	ND	◆	-WW	1	(30)
*C. japonicus*	ACE84673	GH5_8-CBM10-CBM10	◯□[■•]	■◆[□•]	AWW	1	(17)
*Vibrio*	BAA25188	GH5_8-CBM10-CBM10	ND	◆	-WW	0	(30)
*Cellulosi-microbium*	AEE43708	GH5_8-CBM10-CBM10	◯□, chitosan, chitin	■□	AWW	1	(31)
*Cellulosi-microbium*	AEE43708	GH5_8-CBM10-CBM10	◯□, chitosan, chitin	■□	AWW	1	(31)
*Streptomyces*	WP_030268297	CBM10-GH134	[□◯▲, chitin]	■◆	AWW	1	(32, 33)
*Streptomyces*	ADK91085	GH5_8-CBM10	ND	■◆[•▲]	-WW	1	(34)
*St. lividans*	AAA26710	GH5_8-CBM10	ND	■□◆	-WW	1	(35, 42)

ND, not determined.

The order of the proteins follows the grouping seen in the phylogenetic tree (Fig. 3). When an enzyme has more than one CBM10, if possible, the information regarding carbohydrate binding is given for the particular CBM10 (underlined). Biochemical information is given using the following symbols for the polysaccharides: ■, soluble galactomannan; □, insoluble mannan (INM or β-mannan); ◆, soluble glucomannan; •, soluble cellulose (HEC, CMC); ◯, insoluble cellulose (Avicel, BMCC, PASC, filter paper, bagasse); ▲, other soluble polysaccharides (including xyloglycans, xylan, β-glucans, and lichenan). [ ] indicates that the given polysaccharide has been shown not to bind or being a substrate.

CBM10 is a type A surface-binding module with a characteristic “planar” face containing aromatic residues capable of binding to regularly organized surfaces, for example, of the crystalline polysaccharides, cellulose and chitin (36). In the case of CBM10, a typical binding motif has three regularly spaced aromatic side chains, tyrosine, tryptophan, and tryptophan, initially identified in a CBM10 from xylanase A from *Cellvibrio japonicus* (*Cj*_GH10_CBM10) (16, 37) (Table 1). These residues were pivotal for binding to microcrystalline cellulose (Avicel). Furthermore, *Cj*_GH10_CBM10 bound to bacterial microcrystalline cellulose (BMCC) and phosphoric acid–swollen cellulose (PASC) with a dissociation constant (*K*_*d*_) of 0.2 to 0.3 μM but not to hydroxyethyl cellulose (HEC) (15). It moreover bound differentially to plant cell walls depending upon cell type, tissue, and plant taxon (38). *Cj*_GH10_CBM10 is the only structure determined CBM10 (Protein Data Bank ID: 1E8R) (37). Another cellulose-specific *C. japonicus* CBM10 (*Cj*_AA10_CBM10) from LPMO (of auxiliary activities family 10; AA10A) showed a similar polysaccharide-binding preference and bound with *K*_*d*_ = 7.5 to 17.3 μM to disordered (PASC) as well as to crystalline (Avicel and BMCC) cellulose (20), which were also substrates of the LPMO (24). Finally, CBM10 (*Cj*_GH74_CBM10) of the *C. japonicus* GH74 endo-xyloglucanase that also contains a CBM2, when analyzed as a GFP fusion did not bind to soluble tamarind seed xyloglucan and barley 1,3;1,4-β-glucan, both of which are substrates of the GH74 endo-xyloglucanase, or to HEC (Table 1), but bound with high affinity (*K*_*d*_ = 1.5 μM) to Avicel, which is not a substrate (19).

The single CBM10 from a mannanase of GH family 5 subfamily 8 (GH5_8) from the probiotic bacterium *Bifidobacterium animalis* subsp. *lactis* Bl-04 (*Ba*_GH5_CBM10) was able to bind to low-viscosity carob galactomannan (CGM-lv) with a *K*_*d*_ of 0.31 mg/ml but not to microcrystalline cellulose (Avicel), soluble cellulose (HEC), or insoluble ivory nut mannan (INM) (18) (see Fig. 1 for polysaccharide structures) (Table 1). Notably, even though the single *Ba*_GH5_CBM10 was critical for binding onto galactomannan, the enzyme kinetic parameters for this substrate did not change when the CBM10 was removed by truncation, whereas the truncated enzyme was not able to bind galactomannan (18). The lack of binding onto the crystalline cellulose and INM was speculated to be due to substitution of an otherwise conserved tyrosine residue from the typical type A planar carbohydrate-binding motif (16) and also by insertion of five residues, which expand a loop. These characteristics were seen for a phylogenetic subgroup distinct from the classical CBM10s (18).

A CBM10 from *Clostridium cellulolyticum* cellulase of GH9 adsorbed to PASC, Avicel, alkaline-pretreated sugarcane bagasse, and filter paper powder (39). In addition, by comparing 40 type A CBMs (of CBM1, CBM2, CBM3, CBM10, CBM63, CBM64, CBM78, and CBM79) fused with GFP, three CBM10s in two multimodular enzymes from *Teredinibacter turnerae* (GH5_2-CBM5-CBM10-GH6_4) and the marine bacterium *Saccharophagus degradans* (CBM2-CBM10-GH5_4) as well as the *Cj*_GH10_CBM10 mentioned previosuly showed low affinity for Sigmacell cellulose type 20 (20 μm particles) and chitosan compared with the other type A CBMs (21). Finally, CBM10 in a GH134 β-1,4-mannanase from *Streptomyces* sp. NRRL both inhibited enzyme activity and conferred stability at neutral-to-alkaline pH as well as in the presence of organic solvents and detergents (33).

Here, we shall dissect the function of individual CBM10s in a GH5_8 endo-β-mannanase from the marine bacterium and carbohydrate super degrader *S. degradans* by producing the CBM10 domains individually and together as GFP fusions and the full-length enzyme (*Sd*GH5_8-CBM10x3) as well as its three CBM10 truncations (Fig. 2). The three consecutive CBM10s situated C-terminally to the CD are here named *Sd*_GH5_CBM10-1, *Sd*_GH5_CBM10-2, and *Sd*_GH5_CBM10-3 and shown to belong to three distinct phylogenetic subgroups (Fig. 3). Polysaccharide binding was monitored by affinity gel electrophoresis (AGE) or pull-down assays. Enzyme activity was determined using CGM-lv and high-viscosity CGMs and glucomannan, a highly decorated very viscous guar gum (GG) galactomannan, insoluble crystalline INM, and microcrystalline cellulose (Avicel). The three *Sd*_GH5_CBM10s showed distinctly different polysaccharide-binding profiles and impact on the enzyme activity and substrate specificity.

**Figure 2:**
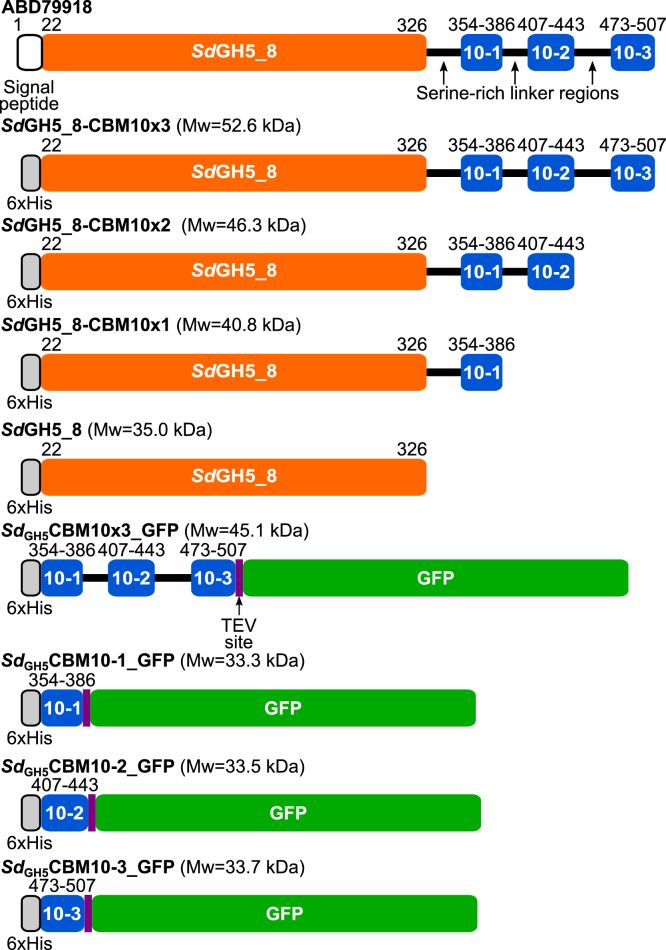
**Domain architecture of the native *Saccharophagus degradans* protein (GenBank accession no.: ABD79918) and the different recombinant full-length and truncated enzyme forms and CBM10 domains produced and characterized.** The residues at domain borders are numbered.

## Results and discussion

### Phylogenetic analysis of CBM10s

**Figure 3:**
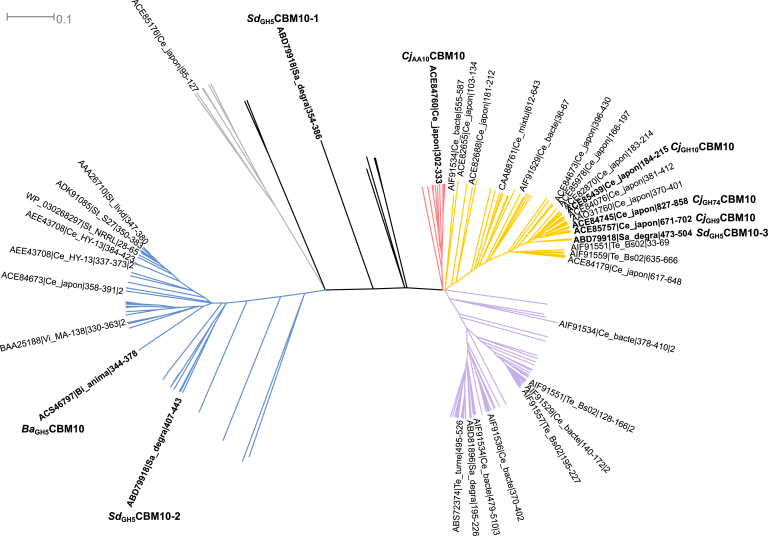
**Phylogenetic tree including CBM10(s) isolated from full-length enzyme sequences (*i.e.*, without CD and other modules).** CBM10s originating from characterized enzymes are labeled (see Table 1 for detailed information). See Figure S1 for the multiple sequence alignment used to generate the phylogenetic tree.

**Figure 4:**
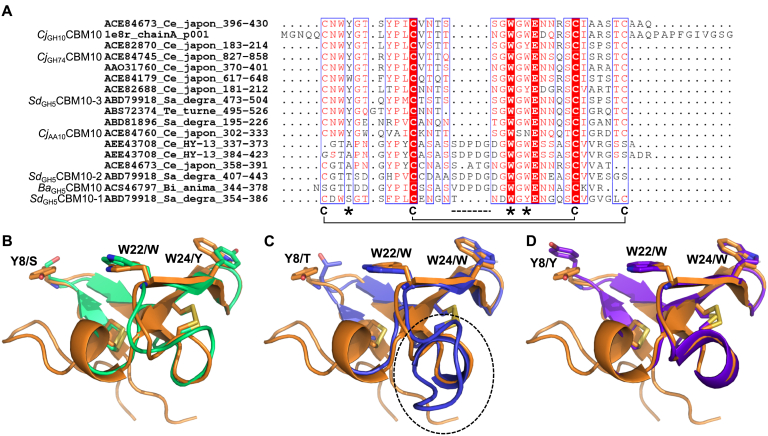
**Comparison of characterized CBM10s.***A*, structure-based multiple sequence alignment including characterized CBM10s. Cysteines involved in disulphide bridges in the *Cj*_GH10_CBM10 structure (Protein Data Bank ID: 1E8R) are denoted by a “C,” whereas binding residues of *Cj*_GH10_CBM10 (Y8, W22, and W24) are indicated by *asterisks*. The insertion differentiating the subgroup containing *Sd*_GH5_CBM10-2 from the other regions of the phylogenetic tree is indicated by a *dotted line*. *B*–*D*, superimposition of the structure of *Cj*_GH10_CBM10 (*orange*; Protein Data Bank ID: 1E8R) and homology models of the three *Sd*_GH5_CBM10s (B. *Sd*_GH5_CBM10-1, *green*; C. *Sd*_GH5_CBM10-2, *blue*; D. *Sd*_GH5_CBM10-3, *purple*) with binding residues shown as s*ticks*. The numbering is according to *Cj*_GH10_CBM10, and the equivalent residue of the *Sd*_GH5_CBM10s is given. The region of the insertion in *Sd*_GH5_CBM10-2 (*C*) is encircled.

The phylogenetic analysis of CBM10 reveals a number of different subgroups (Fig. 3), which have characteristic features with regard to (i) the presence of three aromatic residues (tyrosine, tryptophan, and tryptophan) seen in a canonical type A CBM10 (16), (ii) the presence of cysteine residues potentially forming one of the two observed disulphide bonds, and (iii) the presence of a five to six residues insert between the tyrosine and the two tryptophans of the planar-binding motif (Figs. 4 and S1). For example, the *Ba*_GH5_CBM10 in the *Bifidobacterium* mannanase, which was needed for binding to galactomannan, but did not confer binding to cellulose or INM, had the following distinct features: (i) substitution of the first tyrosine (to a threonine) of the three aromatic residues critical for Avicel binding as demonstrated for *C. japonicus Cj*_GH10_CBM10 (16), (ii) lack of two cysteines that form one of the two disulphide bridges, and (iii) a five-residue long insertion (Figs. 4 and S1).

To further investigate functional characteristics of CBM10s from different regions of the phylogenetic tree, we selected a putative GH5_8 mannanase from the marine bacterium and carbohydrate super degrader *S. degradans* having three C-terminal CBM10s (*Sd*GH5_8-CBM10x3; GenBank accession no.: ABD79918) (Fig. 2). These CBM10s were hypothesized to display distinct functions as they belong to three different phylogenetic subgroups (Fig. 3).

Notably, the innermost *Sd*_GH5_CBM10-1 linked to the CD of the *S. degradans* mannanase (Fig. 2) was found in the phylogenetic tree at a distance of characterized CBM10s. The middle domain, *Sd*_GH5_CBM10-2, however belonged to the same subgroup as *Ba*_GH5_CBM10 from the *Bifidobacterium* mannanase, although they were not closely related. The C-terminal *Sd*_GH5_CBM10-3 belongs to the same subgroup as the previously well-characterized *C. japonicus* CBM10s from xylanase A of GH10 (*Cj*_GH10_CBM10) and of the GH74 endoglucanase active on xyloglucan and mixed-linked 1,3;1,4-β-glucan (*Cj*_GH74_CBM10) (19) (Fig. 3).

### Effect of CBM10s on specificity and activity of GH5_8 endo-β-mannanase

The full-length enzyme, *Sd*GH5_8-CBM10x3, and its three stepwisely truncated forms lacking one, two, or all three CBM10s were recombinantly produced in *Escherichia coli* (Fig. 2). For production of the individual CBM10s and all three CBM10s together, the GFP fusion strategy reported previously to enable production of CBM10 from the GH74 endo-xyloglucanase of *C. japonicus* (*Cj*_GH74_CBM10) (19) was adopted (Fig. 2). All recombinant enzyme forms and the CBM10–GFP fusions were obtained in reasonably good yields of 0.7 to 7.5 mg/g cells after purification. Notably, some of the multimodular forms, in particular the full-length *Sd*GH5_8-CBM10x3, undergo significant proteolytic cleavage in the 21- to 30-residue long serine–glycine rich linker regions during production and purification, which is reflected in the lower yields.

The full-length *Sd*GH5_8-CBM10x3 and its three truncated forms were active on all tested mannan polysaccharides but not on microcrystalline cellulose (Avicel) (Table 2). The substrate specificity of *Sd*GH5_8-CBM10x3 is similar to that of other characterized GH5_8 mannanases but with specific activities at the higher end (17, 18). Removal of the C-terminal CBM10 (*Sd*_GH5_CBM10-3) did not essentially affect the specific activity toward the good substrates, that is, CGM-lv and high-viscosity CGM (locust bean gum) and konjac glucomannan (KGM) (Table 2; Fig. 1). Remarkably, however, removal of the C-terminal *Sd*_GH5_CBM10-3 resulted in doubling, respectively, almost quadrupling of the specific activity (Table 2) on the poor substrates; highly viscous GG galactomannan with on average every second mannosyl backbone unit substituted by galactose (as opposed to one out of four in CGM) and the insoluble crystalline INM (Fig. 1). Removal of both the middle (*Sd*_GH5_CBM10-2) and the C-terminal (*Sd*_GH5_CBM10-3) domains reduced activity by 36% to 40% for CGM-lv and high-viscosity CGMs and maintained activity for KGM compared with the full-length enzyme (Table 2). Remarkably, the activity of *Sd*GH5_8-CBM10x1 with only the innermost CBM10, increased fourfold and ninefold on the poor substrates GG and INM, respectively, compared with the full-length enzyme (Table 2). Finally, removing all three CBM10s essentially recovered activity of the full-length enzyme on CGMs and KGM. This finding resembles the lack of effect on activity for CGM-lv by removing the single C-terminal CBM10 from the *Bifidobacterium* GH5 mannanase (18). Again on the poor substrates GG and INM, the CD alone (*Sd*GH5_8) showed twofold and sixfold higher activity than full-length enzyme. Overall, this suggests that the presence of only *Sd*_GH5_CBM10-1 slightly hinders CGMs from interacting fully effectively with the CD. The presence of the C-terminal and middle CBM10 seems to prevent optimal productive interaction between the CD and the poor substrates INM and the highly substituted guar GG as reflected in the CD alone (*Sd*GH5_8) and the enzyme form with only the first CBM10 (*Sd*GH5_8-CBM10x1) having higher specific activities at low enzyme concentrations.

**Table 2:** Specific activity of full-length SdGH5_8-CBM10x3 and the three truncated forms on soluble (CGM-lv, high-viscosity CGM, KGM, and GG) and insoluble (INM) mannose-containing polysaccharides and the insoluble Avicel (see Fig. 1 for polysaccharide structures)

Substrates	Units	*Sd*GH5_8-CBM10x3	*Sd*GH5_8-CBM10x2	*Sd*GH5_8-CBM10x1	*Sd*GH5_8 (CD alone)
CGM-lv	U/mg	1972 ± 80	2285 ± 150	1539 ± 88	2906 ± 53
	(μmol/s)/μmol protein^a^	1729 ± 70	1763 ± 116	1046 ± 60	1690 ± 31
	Relative (%)^b^	100	102	60	98
CGM (high viscosity)	U/mg	2212 ± 78	2755 ± 109	1814 ± 292	3151 ± 304
	(μmol/s)/μmol protein^a^	1939 ± 69	2126 ± 84	1234 ± 198	1833 ± 177
	Relative (%)^b^	100	110	64	95
KGM	U/mg	3544 ± 110	3617 ± 112	5136 ± 128	4556 ± 108
	(μmol/s)/μmol protein^a^	3107 ± 97	2791 ± 87	3493 ± 87	2650 ± 63
	Relative (%)^b^	100	90	112	85
GG	U/mg	40 ± 8	24 ± 1	200 ± 33	108 ± 25
	(μmol/s)/μmol protein^a^	35 ± 7	19 ± 1	136 ± 23	63 ± 14
	Relative (%)^b^	100	54	389	180
INM	U/mg	9 ± 1	38 ± 3	106 ± 6	81 ± 7
	(μmol/s)/μmol protein^a^	8 ± 1	29 ± 2	72 ± 4	47 ± 4
	Relative (%)^b^	100	363	900	588
Avicel	U/mg	ND^c^	ND^c^	ND^c^	ND^c^

ND, not determined.

a Specific activity calculated based on molecular weight.

b Specific activity relative to full-length *Sd*GH5_8-CBM10x3 calculated based on the specific activity given in (μmol/s)/μmol protein.

c No activity detected.

The kinetic parameters for full-length *Sd*GH5_8-CBM10x3 and truncated forms toward CGM-lv are in agreement with the findings on specific activity (Table 3). Thus, *K*_*m*_ of *Sd*GH5_8-CBM10x1 was threefold higher than of the full-length enzyme, whereas the turnover number increased by 1.8-fold, resulting in 36% lower catalytic efficiency (*k*_cat_/*K*_*m*_). Notably, the catalytic efficiency of CD alone is doubled and 1.4-fold higher than of the *Sd*GH5_8-CBM10x1 and full-length enzyme, respectively. The *K*_*m*_ of CD alone is low and similar to that of full-length *Sd*GH5_8-CBM10x3, whereas the turnover number is 68% higher. The lowest turnover number is seen for full-length enzyme, whereas *Sd*GH5_8-CBM10x1 has the highest turnover number (Table 3).

**Table 3:** Kinetic parameters for full-length SdGH5_8-CBM10x3 and truncated forms on CGM-lv

Enzyme form	*k*_cat_ (s^−1^)	*K*_*m*_ (mg/ml)	*k*_cat_/*K*_*m*_ (ml [mg s]^−1^)
*Sd*GH5_8-CBM10x3	2333 ± 55	2.1 ± 0.1	1096 ± 71
*Sd*GH5_8-CBM10x2	3589 ± 278	4.2 ± 0.7	855 ± 149
*Sd*GH5_8-CBM10x1	4253 ± 337	6.1 ± 0.9	701 ± 114
*Sd*GH5_8	3440 ± 75	2.4 ± 0.1	1413 ± 82

A negative effect on activity of the presence of a CBM10 was previously observed for the GH134 β-1,4-mannanase that contains a single CBM10 and for which the catalytic efficiency of the CD alone on galactomannan was 2.6-fold higher than of the full-length enzyme, whereas *K*_*m*_ was 1.4-fold lower (33), suggesting that the CBM10 is associated with nonproductive binding. This CBM10 did not confer the binding to microcrystalline cellulose, β-mannan, chitin, or xylan as the CD alone can bind to these polysaccharides (33). Although it is not generally seen, there are examples from other CBM families where CBMs have an inhibitory effect on enzyme activity. Thus, removal of a cellulose-binding CBM3 from an endo-β-1,4-glucanase from *Bacillus subtilis* resulted in both increased *k*_cat_ and catalytic efficiency and also improved thermal stability (40). Furthermore, when a CBM5 was removed from a *Pectobacterium chrystantemi* endoglucanase, *K*_*m*_ and *V*_max_ were reduced by 4.5-fold and increased by 2.5-fold, respectively (41).

**Figure 5:**
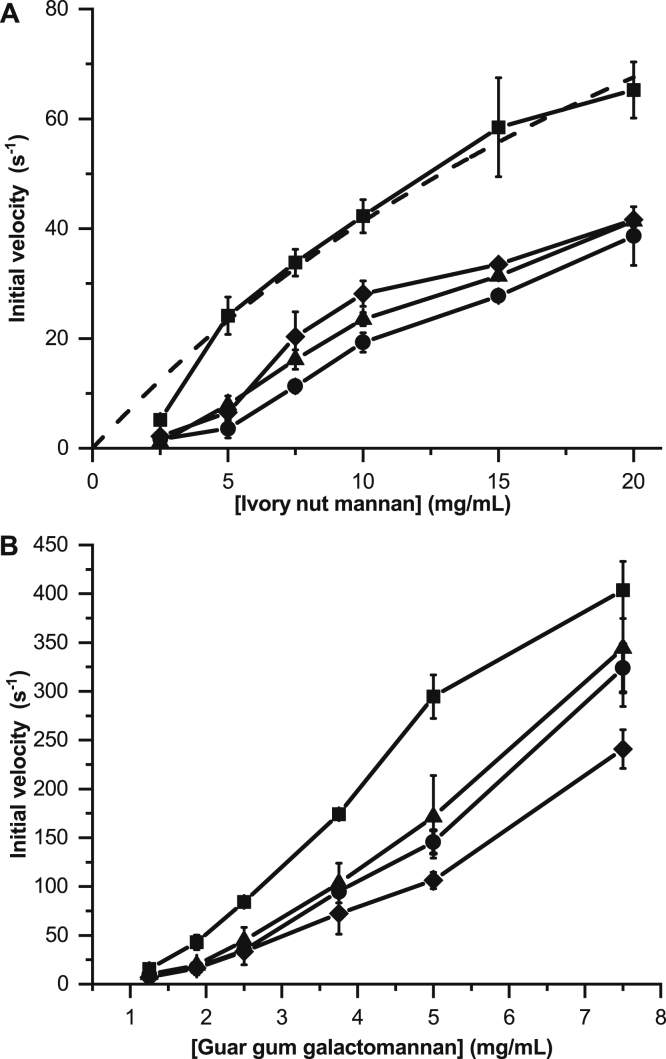
**Kinetics of the full-length enzyme *Sd*GH5_8-CBM10x3.** The three stepwise truncated forms on ivory nut mannan (INM) (*A*) and guar gum galactomannan (GG) (*B*). *Sd*GH5_8-CBM10x3 (■), *Sd*GH5_8-CBM10x2 (•), *Sd*GH5_8-CBM10x1 (▲), and *Sd*GH5_8 (◆). The Michaelis–Menten fit to the *Sd*GH5_8-CBM10x3 data is shown as *dashed line* in *A*.

The kinetics analysis on the insoluble crystalline INM showed the C-terminal CBM10-3 of full-length *Sd*GH5_8-CBM10x3 from *S. degradans* to be needed for maximum activity, whereas the three stepwisely CBM10 truncated enzyme forms showed similarly reduced activity relative to the full-length enzyme (Fig. 5*A*). In the case of GG, the full-length enzyme showed the highest activity, whereas the CD alone in fact was the least active form (Fig. 5*B*). These results on the highly viscous and densely decorated guar GG indicated that the C-terminal CBM10-3 together with CBM10-1 play a role for activity at high-substrate concentrations. Thus, removal of CBM10-3 resulted in some reduction in activity, whereas removal of CBM10-2 as well did not make a difference, as the *Sd*GH5_8-CBM10x1 and *Sd*GH5_8-CBM10x2 showed the same activity. But the removal of CBM10-1 led to a further reduced activity. Although full substrate saturation was not possible, calculations applying the Michaelis–Menten equation on activity of the full-length *Sd*GH5_8-CBM10x3 on INM indicated *K*_*m*_ = 35 ± 14 mg/ml and *k*_cat_ = 186 ± 53 s^−1^ (*k*_cat_/*K*_*m*_ = 5.3 ml/[mg · s]). The *K*_*m*_ is 1.3-fold higher as compared with a GH5_8 mannanase with one CBM10 from *Streptomyces lividans* (*K*_*m*_ = 26.5 ± 11.5 mg/ml), whereas the *k*_cat_ of that enzyme was 69 ± 1.2 s^−1^ resulting in a catalytic efficiency of 2.6 ml/(mg s) (42); hence, *Sd*GH5_8-CBM10x3 is twofold more efficient. Furthermore, the kinetics on INM of the *S. lividans* mannanase was not affected by removal of the CBM10 (42). This CBM10 is in a phylogenetic subgroup with *Sd*_GH5_CBM10-2; hence, the lack of effect on activity on ivory nut is in line with our findings (Fig. 5*A*).

To understand the reason behind the differences observed between the results of the enzyme kinetics on INM and guar GG (Fig. 5) and the specific activity analyses (Table 2), one should have the differences in assay setup in mind. The specific activities were determined as is typical for mannanases at readily manageable low substrate concentrations to avoid highly viscous solutions and maintained (low) enzyme concentration with end point measurement implying substrate-dependent incubation time (18) (Table 2). The kinetics analysis contrarily was done at up to fourfold higher substrate and 10-fold to 30-fold higher enzyme concentration. The action on the insoluble INM should probably be considered as heterogenous catalysis as found in case of cellulases acting on crystalline cellulose, characterized by the presence of different categories of binding sites and productive enzyme attack sites (43, 44). A number of crystalline forms are reported for INM (7, 8), but details on actual mannanase degradation of these forms have not been reported. It is possible that most strongly binding, albeit not productively binding, substrate sites are interacting with full-length enzyme *via* the C-terminal CBM10-3, which deprives the CD access to susceptible substrate bonds. At the higher enzyme concentration used for the kinetics analysis (Fig. 5*A*), this is in part overcome. For the other poor substrate guar GG, the substrate is assumed to contain enzyme target bonds placed in different structural contexts and having different preferences for the productive enzyme attack to occur (6). Although a GH26 mannanase from *B. subtilis* is reported with preference for the guar GG over less substituted galactomannans (45), this is not common, and we assume that initial hydrolysis on GG is slow and may open up for more accessible substrate regions possibly influenced by the fine structural preferences of the CBM10-3 and the CD. This type of action may be observed as a degradation involving apparent cooperative events also indicated by the sigmoidal curvature of the kinetic data (Fig. 5*B*).

### Polysaccharide-binding analysis

The affinities for soluble CGM-lv, HEC, insoluble microcrystalline cellulose (Avicel), and INM were assessed by retardation in AGE and pull-down assays, respectively. Neither the different enzyme forms nor the CBM10s individually or all three CBM10s together were able to bind HEC in AGE. By contrast, both full-length enzyme *Sd*GH5_8-CBM10x3 and the three CBM10s together (*Sd*_GH5_CBM10x3) showed high affinity for CGM-lv (Table 4). Although each of the three *Sd*_GH5_CBM10s when tested individually were able to bind CGM-lv with reasonable affinity, the strong binding depended on the C-terminal domain (*Sd*_GH5_CBM10-3) but was also clearly a combined effect of all the three CBM10s. The full-length enzyme and the three CBM10s together thus bound very strongly with *K*_*d*_ <0.13 mg/ml, whereas neither of the truncated enzyme forms lacking one, two, or all three CBM10 domains showed significant retardation in AGE. Both the C-terminal CBM10-3 and the middle CBM10-2 domains, however, bound reasonably well to galactomannan, the data suggesting that they together secures the full-length enzyme binding. This is opposed to the analysis of the innermost *Sd*_GH5_CBM10-1_GFP, which showed ninefold to 18-fold reduced affinity compared with the other two CBM10s having *K*_*d*_ of 0.4 and 0.2 mg/ml, respectively (Table 4). The second domain (*Sd*_GH5_CBM10-2_GFP), which has an insert of five amino acid residues and lacks a potentially stabilizing disulphide bridge similar to *Ba*_GH5_CBM10 from the *Bifidobacterium* mannanase (Figs. 4 and S1), had a *K*_*d*_ similar to that of *Ba*_GH5_CBM10 (*K*_*d*_ = 0.3 g l^−1^) (18) that it also resembles phylogenetically (Fig. 3). But surprisingly, *Sd*_GH5_CBM10-2_GFP had ninefold higher affinity than the *Sd*_GH5_CBM10-1_GFP (Table 4). A possible explanation is that *Sd*_GH5_CBM10-1, although it probably contains the two stabilizing disulphide bridges, has a serine replacing the first of the three aromatic binding residues and a tyrosine replacing the tryptophan at the position of the third binding site residue (Table 1; Fig. 4). It belongs to a phylogenetic region shared by few CBM10s. Notably, the LPMO *Cj*_AA10_CBM10 also has the third tryptophan replaced (by asparagine) but still has good affinity for insoluble cellulose; however, it does have the conserved tyrosine as the first of the three binding residues (Table 1; Fig. 4).

**Table 4:** Binding to CGM-lv analyzed by AGE

Protein form	*K*_*d*_ (mg/ml)
*Sd*GH5_8-CBM10x3	<0.125^a^
*Sd*GH5_8-CBM10x2	*No binding*
*Sd*GH5_8-CBM10x1	*No binding*
*Sd*GH5_8 (no CBMs)	*No binding*
*Sd*_GH5_CBM10x3_GFP	<0.125^a^
*Sd*_GH5_CBM10-1_GFP	3.7
*Sd*_GH5_CBM10-2_GFP	0.4
*Sd*_GH5_CBM10-3_GFP	0.2
GFP	*No binding*

a Saturation was reached already at the lowest in-gel galactomannan concentration.

**Figure 6:**
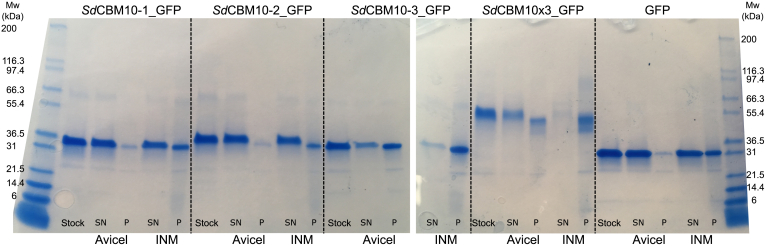
**Qualitative pull-down assay with *Sd***_**GH5**_**CBM10–GFP fusions of each of the three domains *Sd***_**GH5**_**CBM10-1, *Sd***_**GH5**_**CBM10-2, and *Sd***_**GH5**_**CBM10-3 and all three domains combined *Sd***_**GH5**_**CBM10x3_GFP by the insoluble polysaccharides microcrystalline cellulose (Avicel) and ivory nut mannan (INM), respectively.** SDS-PAGE of samples from supernatants (SN) and pellets (P) as well as the protein stocks used for the pull-down assay. GFP alone is included as a control (*right panel*). Molecular mass values (kDa) of marker proteins are indicated.

**Figure 7:**
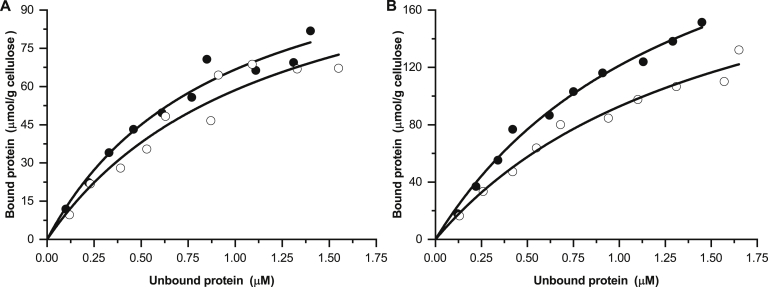
**Quantitative pull-down assay with microcrystalline cellulose (Avicel).** Binding isotherms of the C-terminal domain, *Sd*_GH5_CBM10-3_GFP (*A*) and all three domains together, *Sd*_GH5_CBM10x3_GFP (*B*). The filled and open symbols represent replicates from different days. ﻿No significant binding was observed of the first and the middle domains, *Sd*_GH5_CBM10-1_GFP, *Sd*_GH5_CBM10-2_GFP, or of GFP alone (data not shown).

A qualitative pull down by insoluble INM of the three individual *Sd*_GH5_CBM10 and the combined three domain GFP fusions showed that *Sd*_GH5_CBM10-3_GFP and *Sd*_GH5_CBM10x3_GFP interacted strongly with INM, whereas the amount of protein bound in the case of both *Sd*_GH5_CBM10-1_GFP and *Sd*_GH5_CBM10-2_GFP was in the same range as the amount of GFP alone (Fig. 6). This is in line with the kinetic assay showing that when the C-terminal CBM10-3 was removed, the activity was reduced (Fig. 5*A*). Moreover, the qualitative pull down by Avicel showed only weak interaction with *Sd*_GH5_CBM10-1_GFP and *Sd*_GH5_CBM10-2_GFP, whereas the three CBM10s together (*Sd*_GH5_CBM10x3_GFP) and the C-terminal domain *Sd*_GH5_CBM10-3_GFP bound to Avicel (Fig. 6). Quantitative binding isotherms gave *K*_*d*_ for *Sd*_GH5_CBM10x3_GFP and *Sd*_GH5_CBM10-3_GFP of 1.04 ± 0.16 and 1.48 ± 0.12 μM, respectively (Fig. 7). The maximum amount of bound *Sd*_GH5_CBM10x3_GFP and *Sd*_GH5_CBM10-3_GFP was 0.13 ± 0.00 and 0.27 ± 0.04 μmol/g Avicel, respectively. The binding of *Sd*_GH5_CBM10-1_GFP and *Sd*_GH5_CBM10-2_GFP (and GFP alone) was too weak to obtain a *K*_*d*_. Hence, *Sd*_GH5_CBM10-3 is responsible for Avicel binding, in accordance with findings for other members of the same phylogenetic subgroup (Fig. 3), which had essentially the same affinity as reported also for *Cj*_GH74_CBM10-GFP of GH74 endo-xyloglucanase (19). Notably, the first and second CBM10s in the *S. degradens* mannanase belong to different phylogenetic subgroups and lacked two and one, respectively, of the three residues in the planar aromatic binding motif (16) (Figs. 3 and 4). Previously, it was suggested that the absence of a potentially stabilizing disulphide bond explained a similar lack of Avicel binding for the *Bifidobacterium* full-length mannanase with one CBM10 (18), but this cannot explain why *Sd*_GH5_CBM10-1 is not binding to Avicel, as it has the four cysteines predicted to form the two stabilizing disulphides (Fig. 4). While *Sd*_GH5_CBM10-3 has a *K*_*d*_ for Avicel in the same range as the rest of the characterized CBM10s from the same subgroup in the phylogenetic tree (Fig. 3), it differs by showing ability to bind soluble galactomannan in distinction also from the two other CBM10s from this subgroup tested for galactomannan binding (17).

### Functional diversity of CBM10s

Based on the present results, the C-terminal CBM10, *Sd*_GH5_CBM10-3, is proposed to anchor the secreted enzyme to mannans and to crystalline cellulose, which seems valuable for a free-living marine bacterium as *S. degradans*. *S. degradans* is a so-called super degrader and can utilize complex polysaccharides of algal, higher plant, fungal, and animal origin as sole carbon and energy source (46). Avicel is neither a substrate for *Sd*GH5_8-CBM10x3 not for the *C. japonicus* GH74 endo-xyloglucanase although the CBM10s of both enzymes bind well to Avicel (19). It is well known that CBMs can interact with polysaccharides like cellulose in the plant cell wall or other biomass, rather than soluble branched polysaccharides such as galactomannan (47). However, *Sd*_GH5_CBM10-1 and *Sd*_GH5_CBM10-2 seem to have evolved to become dynamic domains securing flexible searching for an enzyme attack site on a substrate surface or gel for the CD, while being firmly anchored *via* the terminal *Sd*_GH5_CBM10-3. Notably, in particular, *Sd*_GH5_CBM10-2 has reasonable affinity for galactomannan and may potentiate binding to the actual substrate polysaccharide molecule in a structurally complex plant cell wall environment. CBM10 family thus includes members with promiscuous functions binding to a diversity of polysaccharides, such as soluble galactomannan, insoluble crystalline mannans, and cellulose with good affinity (*e.g.* *Sd*_GH5_CBM10-3), whereas some bind soluble polysaccharide substrates with good affinity (*e.g.* *Sd*_GH5_CBM10-2) or with low affinity (*e.g.* *Sd*_GH5_CBM10-1). Members of the same phylogenetic subgroup as *Sd*_GH5_CBM10-3-like CBM10s probably anchor full-length enzymes firmly to substrate or spatially adjacent polysaccharides, whereas CBM10s from phylogenetically different subgroups may still show binding to certain polysaccharides and enable the CD to flexibly survey the vicinity for susceptible glycosidic bonds while yet others may be protein domains serving as linkers or spacers not engaged in direct binding onto a crystalline cellulose *via* a classical CBM type A platform. In nature, multimodular enzymes characteristically participate in heterogenous catalysis on insoluble substrates to which firm binding is mandatory. However, binding is not the same as productive binding, and the density of functional and nonfunctional binding attack sites depends on the material and enzyme as illustrated in details for cellulose degradation by cellulases (43, 44).

## Conclusion and perspectives

The multimodular endo-β-mannanase of family GH5_8 secreted by *S. degradans* is able to bind to polysaccharide substrates and substrate containing biological material by aid of three consecutive C-terminal CBM10 domains. Clearly, the most C-terminal CBM10-3 secures the attachment both to crystalline cellulose (Avicel), galactomannan, and also insoluble seed storage mannan. However, the middle domain CBM10-2 shows also affinity with a 10-fold lower *K*_*d*_ than the *K*_*m*_ for the good substrate galactomannan and may facilitate its interactions with the CD although the truncated enzyme lacking the C-terminal CBM10 lost ability to bind to the galactomannan. The CBM10-2 therefore may also serve as a linker that provides flexibility in polysaccharide interaction for the C-terminal domain and hence for the CD to interact productively with substrate. The role of the innermost CBM10-1 is more enigmatic, but at low enzyme and substrate concentrations, it was able to secure highest activity of the CD toward the two poor substrates (INM and guar GG) also relative to the activity of the CD alone or with two, respectively, three CBM10s. The CBM10-1 is in a small separate phylogenetic subgroup for which a possible hallmark and biological role, beyond this mentioned stabilization, remains to be identified.

## Experimental procedures

### Bioinformatics

CBM10 sequences were retrieved from the CAZy database (www.cazy.org; (10)) and subjected to multiple sequence alignment using MUltiple Sequence Comparison by Log-Expectation tool (48). A phylogenetic tree was constructed using BIONJ (49) and visualized using Dendroscope, version 3.6.3 (https://uni-tuebingen.de/fakultaeten/mathematisch-naturwissenschaftliche-fakultaet/fachbereiche/informatik/lehrstuehle/algorithms-in-bioinformatics/software/dendroscope/) (50). A structure-based alignment of selected characterized CBM10s was generated using the PROMALS3D Web server (51). Homology models of the three *Sd*_GH5_CBM10s were made using HHpred (https://toolkit.tuebingen.mpg.de/tools/hhpred) (52) with the structure of *Cj*_GH10_CBM10 (Protein Data Bank ID: 1E8R) as template.

### Carbohydrates

CGM-lv, INM, and KGM were from Megazyme; high-viscosity CGM (locust bean gum), GG, microcrystalline cellulose (Avicel), and HEC were from Sigma–Aldrich. See Figure 1 for the polysaccharide structures.

### Gene constructs

The genes encoding the *S. degradans* β-mannanase *Sd*GH5_8-CBM10x3 (GenBank accession no.: ABD79918) without the predicted signal peptide (amino acids 1–21) and the three CBM10s *Sd*_GH5_CBM10x3 fused with GFP, and GFP (AGT98536) were purchased (GeneArt; Thermo Fisher Scientific), and cloned into the pET28a(+) vector using the XhoI and NheI restriction sites resulting in an N-terminal cleavable His tag. The three C-terminally truncated forms *Sd*GH5_8-CBM10x2, *Sd*GH5_8-CBM10x1, and *Sd*GH5_8 (Fig. 2) were constructed by introducing stop codons using Q5 Site-Directed Mutagenesis Kit (New England Biolabs), pET28a-*Sd*GH5_8-CBM10x3 as template and mutagenesis primers (Table S1) designed according to the manufacturers' instructions. Each of the three *Sd*_GH5_CBM10s were obtained as *Sd*_GH5_CBM10 to 1_GFP, *Sd*_GH5_CBM10 to 2_GFP, and *Sd*_GH5_CBM10-3_GFP by mutagenesis using Q5 Site-Directed Mutagenesis Kit (New England Biolabs), pET28a-*Sd*_GH5_CBM10x3_GFP as template and mutagenesis primers. To get *Sd*_GH5_CBM10-1_GFP, both *Sd*_GH5_CBM10-2 and *Sd*_GH5_CBM10-3 were deleted in one step. A one-step deletion of *Sd*_GH5_CBM10-1 and *Sd*_GH5_CBM10-2 was done to get *Sd*_GH5_CBM10-3-GFP. Finally, *Sd*_GH5_CBM10-2_GFP was obtained in two steps; first deleting CBM10-1 and then CBM10-3. The gene constructs were confirmed by sequencing.

### Protein production

All full-length and variant-encoding plasmids were transformed into *E. coli.* BL21, screened on LB agar including 50 μg/ml kanamycin, and starter cultures (10 ml) were made by inoculating LB medium, 50 μg/ml kanamycin with a single colony and incubated (37 °C, overnight), which are used to inoculate 750 ml LB medium containing 10 mM glucose and 50 μg/ml kanamycin in shake flasks. Cultures were propagated (30 °C, 160 rpm) to an absorbance of 0.6 at 600 nm followed by decreasing the temperature to 16 °C and induction of expression by a final concentration of 0.1 mM IPTG. Cells were harvested (4000*g*, 20 min, 4 °C) after approximately 20 h and stored at –20 °C until protein purification.

### Protein purification

The proteins were purified in two steps. Cells were resuspended in HisTrap equilibration buffer (10 mM Hepes, pH 7.4, 10 mM imidazole, 0.5 M NaCl, and 10% glycerol), added two cOmplete Mini, EDTA-free, protease inhibitor tablets (Roche Diagnostics GmbH), lysed using a high-pressure homogenizer at 1 bar, added 3 μl Benzonase Nuclease (Sigma–Aldrich), and centrifuged (40,000*g*, 4 °C, 30 min). The filtered (0.45 μm) supernatant was loaded onto a 5 ml HisTrap HP column (GE Healthcare) pre-equilibrated with HisTrap equilibration buffer at a flow of 3 ml/min and eluted by a linear gradient from 2.5% to 100% HisTrap elution buffer (10 mM Hepes, pH 7.4, 400 mM imidazole, 0.5 M NaCl, and 10% glycerol) in 20 column volumes. Protein-containing fractions (based on absorbance at 280 nm) were pooled, concentrated to 4 ml (10 kDa Amicon Ultra centrifugal filters; Merck-Millipore) and further purified by gel filtration (Superdex 16/60 75 equilibrated with 10 mM Hepes, pH 7.0, 150 mM NaCl, and 10% glycerol) at a flow rate of 0.5 ml/min. Fractions containing recombinant protein of correct size as based on SDS-PAGE were pooled and concentrated as above to 2 to 3 mg/ml. The absorbance at 280 nm was measured, and the protein concentration was determined using theoretical extinction coefficients.

### Substrate specificity

The specific activity was determined essentially as previously described (18, 53) using a reducing end 3,5-dinitrosalicylic acid (DNS) assay. Briefly, full-length and truncated enzyme forms (0.4–9.1 nM) were incubated with low- and high-viscosity galactomannans (2.5 mg/ml, 10 min), KGM (2.5 mg/ml, 10 min), GG (2.5 mg/ml, 2.5 h), INM (5 mg/ml, 45 min), or Avicel (10 mg/ml, 3 h) under standard activity assay conditions (400 μl assay volume, 40 mM sodium phosphate citrate pH 6.0, 0.005% [v/v] Triton X-100, 37 °C) in triplicates. The reactions were stopped by addition of 600 μl DNS reagents followed by heat treatment at 95 °C for 15 min. After 15 min in ice water, the samples were centrifuged at 20,000*g* for 12 min. Finally, the absorbance was measured at 540 nm.

### Kinetic analysis

The kinetics on CGM-lv, GG, and INM were determined using the aforementioned DNS assay with a range of substrate concentrations (0.45–9 mg/ml CGM-lv; 1.25–7.5 mg/ml GG; and 2.5–20 mg/ml INM) and enzyme (full-length and truncated forms: 1.5–2.8 nM for CGM-lv, 13–25 nM for GG, and 99–179 nM for INM) in 2000 μl and withdrawing 400 μl aliquots (200 μl in the case of INM) at 3, 6, 9, and 12 min. The kinetic assays were done in duplicates. In the case of the CGM-lv and INM data, the Michaelis–Menten model was fitted to the initial velocity data using GraphPad Prism 6 (GraphPad Software Inc).

### Pull-down assay

Qualitative screening of binding of the individual CBM10s separately and the triple CBM10 to microcrystalline cellulose (Avicel) and insoluble crystalline INM was done by a pull-down assay, where the samples were analyzed by SDS-PAGE: 10 mg polysaccharide (prewashed three times) was mixed with 200 μl 0.5 mg/ml protein in assay buffer (50 mM phosphate buffer, pH 7.0). GFP was used as a control in the assay. The samples were incubated at 4 °C for 1 h with gentle agitation and then centrifuged (20,000*g*, 10 min, 4 °C). Supernatants were transferred to fresh tubes and centrifuged (20,000*g*, 10 min, 4 °C), before 4 μl supernatant was heat treated in the presence of SDS-loading buffer and applied on the SDS-PAGE. The pellets from the pull-down assay were washed with 250 μl assay buffer and pelleted again as aforementioned, resuspended in 200 μl assay buffer, added 50 μl SDS-loading buffer, boiled for 10 min, and applied (4 μl) on the SDS-PAGE. For each protein, a sample of the 0.5 mg/ml protein stock used for the pull-down assay was treated as the supernatant sample and included on the gel.

Quantitative pull-down assay was done with microcrystalline cellulose (Avicel) utilizing the presence of GFP for quantification (19). The total assay volume (750 μl) contained 10 mg/ml Avicel (washed with assay buffer thrice) and the different *Sd*_GH5_CBM10-GFP forms in ten different concentrations (10–100 μg/ml) in 50 mM phosphate pH 7.0 and 0.1 mg/ml bovine serum albumin. GFP (10–150 μg/ml) served as a negative control. The mixtures were rotated end over end (4 °C, 1 h) and centrifuged (20,000*g*, 10 min, 4 °C). Supernatants were transferred to fresh tubes, centrifuged (20,000*g*, 10 min, 4 °C), and the concentration of unbound protein was determined by fluorescence (FP-8500 Spectrofluorometer equipped with an FMP-825 plate reader; JASCO Corporation) with excitation at 450 and emission at 510 nm using a standard curve made with each protein form (1–1250 nM) in assay buffer. The amount of bound protein was calculated by subtracting the unbound from the total protein, and the dissociation constant, *K*_*d*_, was obtained by fitting Equation 1 to binding isotherm data (GraphPad Prism, version 6).

(1) $$\left[\text{PC}\right]=\frac{\left[\text{FP}\right]{\left[\text{PC}\right]}_{\max}}{K_{d}}$$

PC and FP are the bound and unbound protein concentrations, respectively. The pull-down assays were done in duplicates on two different days.

### AGE

Qualitative screening of binding of all protein forms and GFP (as control) was done by AGE with 2.5 mg/ml CGM-lv or HEC in 12% acrylamide gels (54). Protein (10 μl 0.4 mg/ml) in loading buffer was applied to polysaccharide-containing and control (without polysaccharide) gels, respectively. NativeMark unstained protein standard (Invitrogen) was included on all gels for normalization. The gels were run (20 h at 4 °C and 45 V) and stained using InstantBlue (Expedeon). Quantitative protein binding analysis to CGM-lv was done from migration and retardation in gels without (control) or with 0.125, 0.25, 0.5, 1, 1.5, and 2.5 mg/ml CGM-lv, respectively. *K*_*d*_ was determined from retardation distances according to the method (54) using a Takeo–Nakamura plot (55) (Equation 2):

(2) $$\frac{1}{R_{\text{mi}}}=\frac{1}{R_{\text{mo}}}\left(1+\frac{c}{K_{d}}\right)$$

*R*_mi_ and *R*_mo_ are migration distances of sample relative to a reference protein in the presence and absence of polysaccharide, respectively; *c* is the polysaccharide concentration. The relative migration (Equation 3) defines the retardation of migration by the polysaccharide compared with the control.

(3) $$R_{\text{m}}=\frac{R_{\text{mi}}}{R_{\text{mo}}}$$

## Data availability

All the data are contained within the article.

## Supporting information

This article contains supporting information.

## Conflict of interest

The authors declare that they have no conflicts of interest with the contents of this article.
