# Supplementary material for: Functional diversity of three tandem C-terminal carbohydrate-binding modules of a β-mannanase
Source: J Biol Chem. 2021 Apr 7;296:100638. doi: 10.1016/j.jbc.2021.100638 (PMC8121702; doi:10.1016/j.jbc.2021.100638)
Supplement: Table S1 and Figure S1 [file mmc1.pdf]

SUPPORTING INFORMATION

Functional diversity of three tandem C-terminal carbohydrate-binding modules of a β-mannanase

Marie Sofie Møller[1]*, Souad El Bouaballati[1], Bernard Henrissat[2,3] and Birte Svensson[1]

[1]Department of Biotechnology and Biomedicine, Technical University of Denmark, DK-2800 Kgs. Lyngby, Denmark

[2]Architecture et Fonction des Macromolécules Biologiques, CNRS, Aix-Marseille Université, 13288 Marseille, France

[3]Department of Biological Sciences, King Abdulaziz University, Jeddah, Saudi Arabia

*Corresponding author: Marie Sofie Møller
E-mail: msmo@dtu.dk

Table S1. Mutagenesis primers used for obtaining the gene constructs. f, forward primer; r, reverse primer.

**Primer**	**Sequence**	**Melting temperature**
*SdGH*5_8-CBM10x2-f<br>*Sd*GH5_8-CBM10x2-r	5’-AAGCGGTAGCtaaTCGAGTTCATC-3’<br>5’-TCAACGACACAACTGGCT-3’	62°C
*Sd*GH5_8-CBM10x1-f<br>*Sd*GH5_8-CBM10x1-r	5’-AGGTTTATGCtaaTCTAACCCCG-3’<br>5’-ACACCTACACAACTTTGG-3’	58°C
*Sd*GH5_8-f<br>*Sd*GH5_8-r	5’-AGGAACGTCTtgaGGCGGCAGCT-3’<br>5’-GTGTACACACTGGCTATACGC-3’	68°C
*Sd*GH5CBM10-1_GFP-f<br>*Sd*GH5CBM10-1_GFP-r	5’-GAGAATCTTTATTTTCAGGG-3’<br>5’-GCATAAACCTACACCTAC-3’	56°C
*Sd*GH5CBM10-2_GFP-step 1-f<br>*Sd*GH5CBM10-2_GFP-step 1-r	5’-TGCGGCACTACCAGCGAC-3’<br>5’-GCTAGCCATATGGCTGCC-3’	66°C
*Sd*GH5CBM10-2_GFP-step 2-f<br>*Sd*GH5CBM10-2_GFP-step 2-f	5’-GAGAATCTTTATTTTCAGGGCATG-3’<br>5’-GCTACCGCTTTCAACGAC-3’	62°C
*Sd*GH5CBM10-3_GFP-f<br>*Sd*GH5CBM10-3_GFP-r (same as *Sd*GH5CBM10-2_GFP-step 1-r)	5’-TGTAATTGGTATGGCACTCAATAC-3’<br>5’-GCTAGCCATATGGCTGCC-3’	64°C

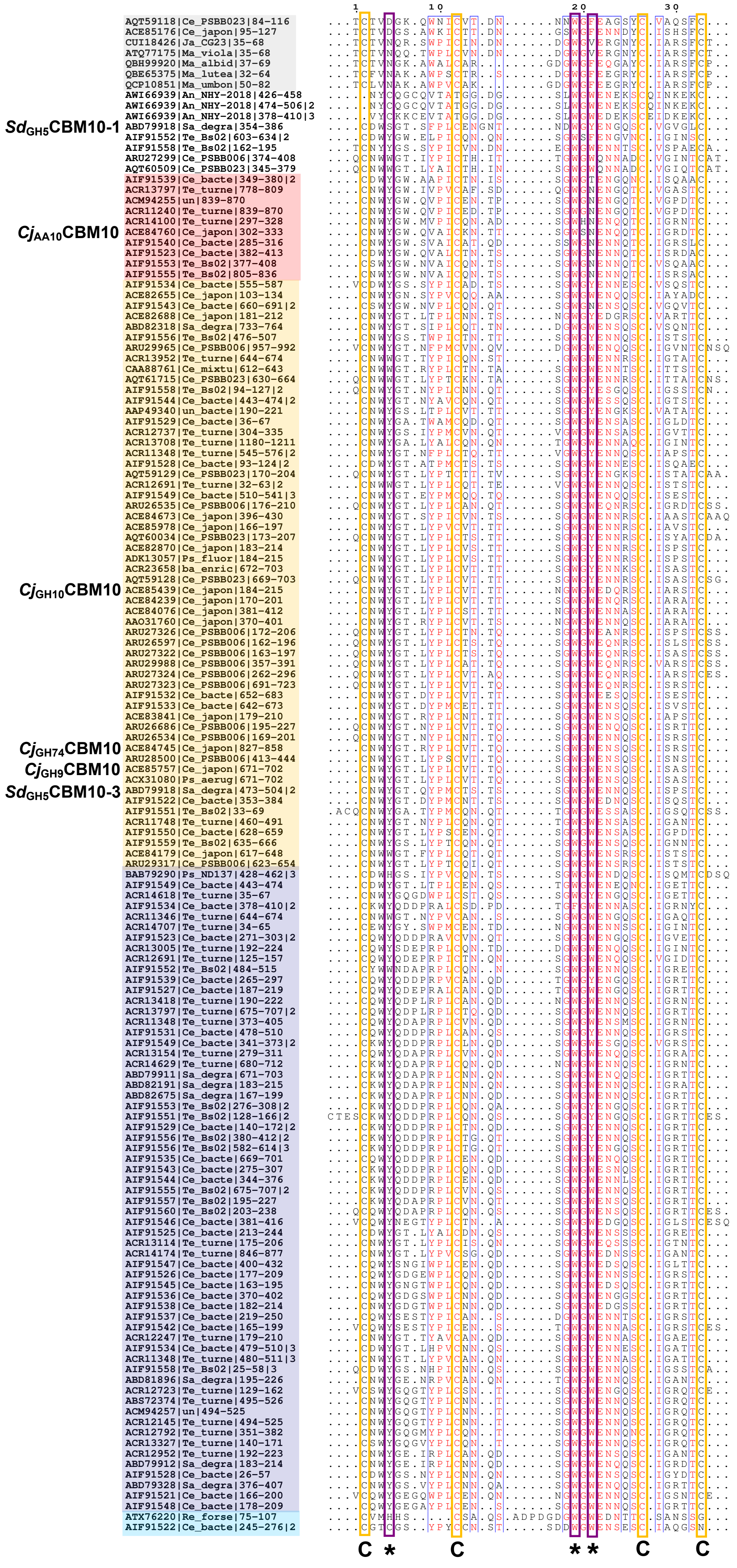
SdGH5CBM10-1
CjAA10CBM10
CjGH10CBM10
CjGH74CBM10
CjGH9CBM10
SdGH5CBM10-3
C * C * * C C

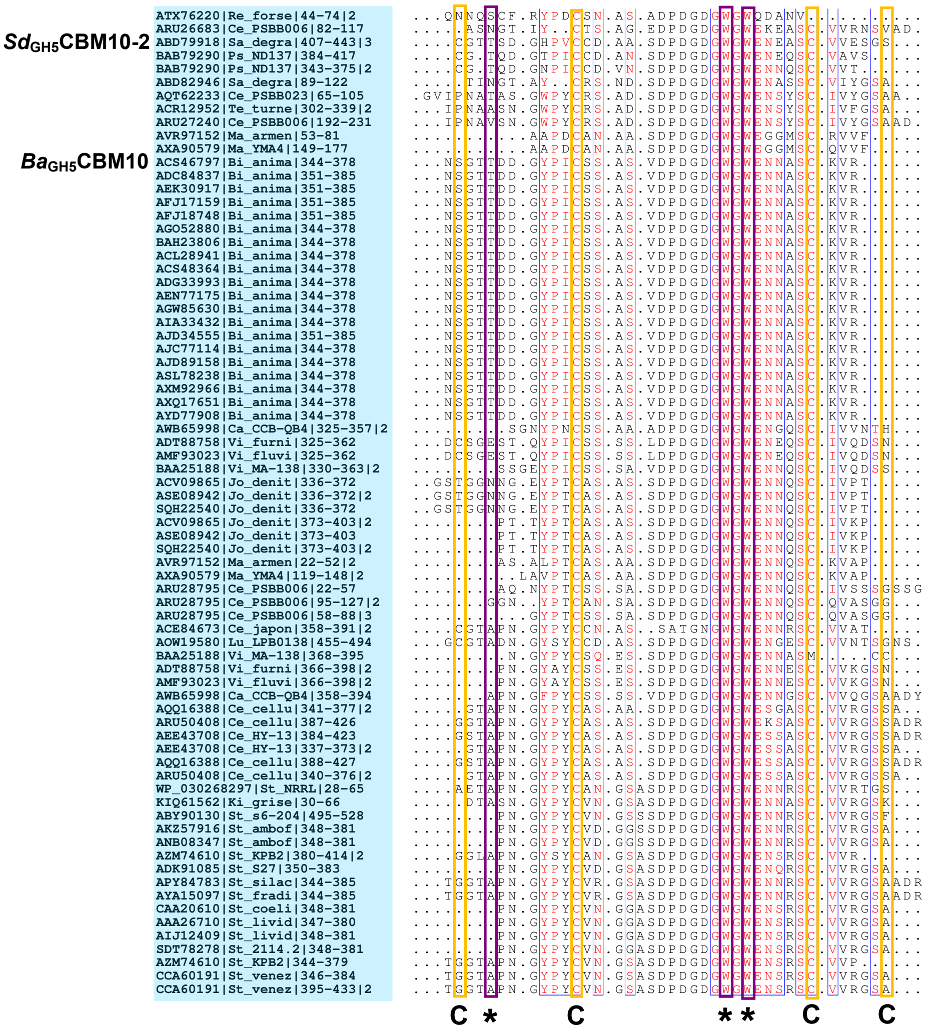

Figure S1 Full multiple sequence alignment used for generating the phylogenetic tree in Figure 2. Cysteines involved in disulphide bridges in the *Cj*$_{GH10}$CBM10 structure (PDB ID 1E8R) are denoted by a “C” (highlighted in yellow), while binding residues of *Cj*$_{GH10}$CBM10 are indicated by asterisks (highlighted in purple).
